# Abstracts/Sommaires/Zusammenfassungen

**DOI:** 10.1155/S1463924679000225

**Published:** 1979


### Abstracts Sommaires Zusammenfassungen

### Page 69

**Specification and production of prototype automatic instrumentation**
J. E. Carlyle

Commercially available instrumentation used in chemical laboratories is frequently developed from prototype units produced by laboratory personnel. This paper outlines the chemical and engineering expertise required and shows the various stages of development. Information needed to allow a feasibility study is outlined and details of dispensing systems, temperature control, mixing, detector systems, and data handling are discussed. Critical path analysis of a detailed production schedule is essential to contain the project within a reasonable development period. Development functions which can be scheduled in parallel are shown and the use of commercially available sub-units is discussed.

**Specification et production de l'instrumentation prototype automatique**

L'instrumentation disponible commercialement, employée dans laboratoires chimiques, fréquemment est développée des unités prototypes produites par personnel du laboratoire. Cet écrit esquisse l'expertise, chimique et du génie, requise et indique les stades variés de développement. L'information necessaire pour faire une étude de faisabilité est esquissée, et les détails des systèmes à dispenser, la commande du température, la mélange, les systèmes à détecteur, et le traitement des informations sont discutés. L'analyse de chemin critique d'un plan détaillé de production est essentielle pour borner le projet à une période raisonnable de développement. On montre les fonctions de développement qu'on peut dresser en parallèle, et discute l'emploi des sous-unités disponibles commercialement.

**Specifizierung und Produktion von urbilden automatischen Instrumentierung**

Laborpersonal entwickeln oft aus Urbildeinheiten, käufliche Instrumentierung für chemische Laboratorien. Diese Schrift umreisst dei nötige chemische und ingenieurwesene Gewandtheit und zeigt die verschiedenen Entwicklungstadien. Die notwendige Auskunft für ein Durchführbarkeitstudium wird umgerissen und die Einzelheiten der Dispensierungsystemen, des Temperaturreglers, der Mischung, der Detektorsystemen und der Datenhandhabung werden diskutiert. Kritische Weganalyse einer ausführlichen Herstellungtabelle ist wesentlich um das Projekt innerhalb eines verständigen Entwicklungzeitabschnitts zu enthalten. Entwicklungfunktionen, die in Parallele tabellarisch zusammenstellen können, werden gezeigt und der Nutzen der käuflichen Untereinheiten wird diskutiert.

### Page 72

**Design features of a computer controlled multiplexed absorptiometer for reaction rate analysis** by M. Snook, A. Renshaw, J. M. Rideout, D. J. Wright, J. Baker and J. Dickens.

The multiplexed absorptiometer described is part of an analytical system for monitoring kinetic reactions in the Clinical Biochemistry laboratory at high sample throughput rates. At a continuous sample throughput rate of 150 tests per hour, the dual wavelength absorptiometer can monitor reactions for 34 minutes. Sample throughput can easily be changed, there being a trade-off between throughput and monitoring period. Data acquisition and instrument control is accomplished by a Texas 960 A minicomputer driven from an external source phase-locked to data acquisition.

The scanning beam absorptiometer monitors a circular array of 100 cuvettes every two seconds. Each cuvette is read at least 10 times at two wavelengths in the 10 mS available to provide sufficient data for signal 'normalisation'. Analogue signal processing reduces this data to a 12 bit Beer's Law corrected data point for subsequent curve fitting. Software subroutines optimise the data acquisition time-base for individual reactions, and make real-time best lines of fit.

**Specifications d'un absorptiomètre controle par ordinateur pour l'analyse cinétique d'un grand nombre d'échantillons**

L'absorptiomètre multiplexé décrit, fait partie d'un système analytique pour le contrôle de réactions cinétiques dans le laboratoire clinique-biochmique à grand débit d'échantillons. Pour une capacité constante de 150 déterminations par heure, l'absorptiomètre à double longueur d'onde peut contrôler des réactions pendant 34 minutes. La capacité de passage peut facilement être variée, car la capacité de passage et le temps de contrôle sont interdépendants. La prise des données et le contrôle des instruments est effectué par un miniordinateur Texas 960 A sinchronisé par un signal externe pour l'acquisition des données.

L'absorptiomètre à longueur d'onde réglable contrôle toute les deux secondes un arrangement circulaire de cent cuvettes. La lecture de chaque cuvette se fait pendant 10 ms à deux longueurs d'onde différentes, afin d'obtenir suffisemment de données pour la normalisation des signaux. Une réduction analogique des signaux donne un point de donnée de 12 bit corrigé selon la loi de Beer. Ce point est ensuite utilisé pour l'adaptation de la courbe. Des subroutines optimisent la base de temps pour le transfer des données des réactions particulières et exécutent l'adaptation de la courbe en temps réel.

**Spezifikationsmerkmale eines rechnergesteurerten Absorptiometers fuer die gleichzeitige Kinetische Analyse vieler Proben**

Das beschriebene multiplexe Absorptiometer ist Teil eines analytischen Systems für die Überwachung kinetischer Reaktionen im klinisch-biochemischen Laboratorium bei gleichzeitig hohem Probendurchsatz. Bei steter Durchsatzrate von 150 Bestimmungen pro Stunde kann das Doppelwellenlägen-Absorptiometer Reaktionen während 34 Minuten überwachen. Der Probendurchsatz kann ohne weiteres verändert werden, weil ein Abtausch zwischen Durchsatz und Länge der Ürwachungsperiode möglich ist. Datenaufnahme und Gerätesteuerung wird von einem Texas 960 A Minicomputer besorgt, der über ein externes Signal auf die Datenübernahme synchronisiert wird.

Das Absorptiometer mit einstellbarer Wellenlänge überwacht alle 2 Sekunden eine kreisförmige Anordnung von 100 Küvetten. Jede Küvette wird während den zur Verfügung stehenden 10 ms mindestens 10 mal bei zwei verschiedenen Wellenlängen abgetastet, um genügend Daten für die Signal-Normalisierung liefern zu können. Analoge Signalverarbeitung reduziert diese Daten zu einem gemäss dem Beerschen-Gesetz korrigierten 12 bit Datenpunkt, der für nachfolgende Kurvenanpassung verwendet werden kann. In der Software optimieren Unterprogramme die Zeitbasis für die Datenübernahme der einzelnen Reaktionen und führen in Echzeit die Kurvenanpassung durch.

### Page 77

**An automatic system for kinetic clinical analyses** by Clifford Riley, Colin F. Darby, David E. Tutt and Bernard F. Rocks.

A fluid handling system intended for emergency analysis in hospitals has been developed. This has the minimum of moving parts and control of movement of liquids is achieved by use of a combination of compressed air and vacuum. Some of the analytical applications of the system are briefly described.

**Un système automatique pour analyses cinétiques dans le laboratoire clinique**

Un système pour manipulation de liquides pour analyses d'urgences dans les hopitaux a été développé. Le mouvement des liquides s'effectue par une combinaison d'air comprimé et de système sous vide. Le système contient un minimum de parts mobiles. Quelques applications analytiques de ce système sont brèvement décrites.

**Ein automatisches System fuer kinetische analysen im klinischen Laboratorium**

Es wurde ein System für System die Hantierung von Flüssigkeiten entwickelt, das für die Benützung bei Eilanalysen in Spitälern vorgesehen ist. Dieses System weist ein Minimum an bewegten Teilen auf und benützt für die Bewegung der Flüssigkeiten eine Kombination von Druckluft und Vakuum. Einige der analytischen Anwendungen dieses Systems werden kurz beschrieben.

### Page 82

**The design of a microprocessor system for automatic signal averaging and percentage purity calculations coupled to a nuclear magnetic resonance spectrometer** by F. Morley, I. K. O'Neill, M. A. Pringuer and P. B. Stockwell.

A microprocessor control system interfaced to a nuclear magnetic resonance spectrometer is described. It enables automatic control of the spectrometer operation, reduced the noise levels by signal averaging and computes the percentage purity of the test materials. Operator intervention is considerably reduced. The design requirements for the integrator and spectrometer system are described along with the microprocessor hardware and software need to construct the automatic system. Quantitative measurements of pharmaceutical preparations are facilitated using this technique.

**Construction d'un système de contrôle par microprocesseur connecté à un spectromètre RMN pour formation de moyenne du signale et pour calculation du degré de pureté**

Un système de contrôle par microprocesseur connecté à un spectromètre RMN est décrit. Cet instrument de contrôle permet l'utilisation automatique du spectromètre, reduit le bruit de fonds par formation de moyenne et calcule le degré de pureté de substances de test. Les interventions par l'utilisateur sont minimes. Les spécifications nécéssaires pour l'intégrateur et le spectromètre sont décrite en connexion avec le microprocesseur et le "software" nécésaire pour la construction du system automatique. Des mesures quantitatives de produits pharmaceutiques sont facilitées par cette technique.

**Konstruktion eines an ein Kernresonanz-Spektrometer angeschlossenen Mikroprozessor-systems fuer automatische signal-mittelung und berechnungen von Reinheitsgraden**

Es wird ein Mikroprozessor-Steuergerät beschrieben das an ein Kernresonanz-Spektrometer angeschlossen ist. Dieses Steuergerät ermöglicht den automatischen Betrieb des Spektrometers, erniedrigt das Rauschen durch Signalmittelung und berechnet den Reinheitsgrad von Testsubstanzen. Die Bedienung durch den Benützer ist stark vereinfacht. Die notwendigen Spezifikationen für den Integrator und das Spektrometer-System werden zusammen mit der für den automatischen Betrieb benötigten Mikroprozessor-Hardware und Software beschrieben. Quantitative Messungen an pharmazeutischen Präparaten werden mit dieser Technik erleichtert.

### Page 88

**An improved automatic liquid injection apparatus for use on a carbon analyser** by R. A. Van Steenderen.

A modification of an earlier injection apparatus is described. It was developed to overcome disadvantages in previous designs and has the further advantage of enabling the determination of total and inorganic carbon in water in the range 10 to 50 mg/dm³ at a rate of 20 samples/h with a standard deviation of less than 1 per cent.

**Un appareil automatique ameliore pour l'injéction de liquides dans un analysateur de carbone**

La modification d'un appareil d'injéction est décrite. Elle a été effectuée pour éliminer les désavantages de constructions antérieures. En plus elle permet la détermination du carbone total et anorganique entre 10 et 50 mg/dm³ avec une capacité de 20 échantillons par heure. La déviation standard est inférieure a 1 pour cent.

**Ein verbessertes automatisches geraet fuer die Fluessigkeitseinspritzung zur verwendung in einem Kohlenstoff-analysator**

Eine Modifikation eines früheren Einspritzgerätes wird beschrieben. Sie wurde durchgeführt um Nachteile früherer Konstruktionen zu beseitigen und weist weiter den Vorteil auf, dass damit die Bestimmung des gesamten und anorganischen Kohlenstoffs in Wasser im Bereich zwischen 10 und 50 mg/dm³ und bei einem Durchsatz von 20 Proben pro Stunde mit einer Standardabweichung von weniger als 1 Prozent ermöglicht wird.

### Page 91

**An automated distillation method for the determination of diacetyle in beer: a comparison of analysis by AutoAnalyzer and gas chromatography** by J. C. Buijten and B. Holm.

A fully automated method for the determination of diacetyl in beer has been developed. The method includes a distillation step. The distillate was mixed with o-phenylenediamine solution and the absorbance of the product, 2,3-dimethyl-chinaxoline, was measured at 340 nm. The accuracy of the distillation method has been assessed by comparison with a head space gas chromatographic method. The correlation coefficient between the two techniques was highly significant, $r = 0.996 \pm 0.0163$ ($n = 25$, $p < 0.001$).

**Une méthode automatique de destillation pour la détermination de diacétyle dans la bière: comparaison avec l'analyse par AutoAnalyzer et par chromatographie en phase gazeuse**

Une méthode complètement automatisée pour la détermination de diacétyle dans la bière a été developpée. Cette méthode inclut un pas de destillation. Le destillat est mélangé avec une solution de o-phénylenediamine et l'absorbance du produit, 2,3-diméthylechinaxoline, est mesuré à 340 nm. La précision de cette méthode est déterminé par comparaison avec la chromatographie en phase gazeuse des "surnageants." La coefficient de corrélation entre les deux techniques est hautement signifiquant, $r = 0.996 \pm 0.0163$ ($n = 25$, $p < 0.001$).

**Eine automatisierte Destillationsmethode fuer die Bestimmung von Diacetyl in Bier: ein Vergleich der Analyse mit Auto-Analyzer und Gaschromatographie**

Eine vollautomatisierte Methode für die Bestimmung von Diacetyl in Bier wurde entwickelt. Diese Methode umfasst einen Destillationsschritt. Das Destillat wurde mit einer Lösung von o-Phenylendiamin vermischt und die Absorption des Produktes, 2,3,-Dimethyl-Chinaxolin, bei 340 nm vermessen. Die Genauigkeit der Destillationsmethode wurde beurteilt durch einen Vergleich mit der Methode der Dampfraum-Gaschromatographie. Der Korrelationskoeffizient zwischen den beiden Techniken zeigt hohe Signifikanz: $r = 0.996 \pm 0.0163$ ($n = 25$, $< 0.001$).

### Page 94

**Clinical laboratory evaluation of the Orion SS-20 Ionized Calcium Analyser** by Patrick Ferreira and Alan M. Bold.

An evaluation of the Orion SS-20 Ionized Calcium Analyser is presented. Data on precision (day-to-day and within-run), linearity and recovery, potential interferences (including red cells), and normal reference values are given. Results are directly compared with those using a spectrophotometric technique. Problems that were encountered included unpredictable day-to-day variations in sensor response to serum samples, loss of $CO_2$ from samples traversing the analyser tubing, and unexplained errors in the first sample or a run. Methods are suggested for overcoming each of these difficulties.

**Evaluation de l'analysteur pour calcium ionisé Orion SS-20 dans le laboratoire clinique**

Une evaluation de l'analysateur pour calcium ionisé ORION SS-20 est présentée. Des donneés sur la précision (dans une serie de mesures et de jour en jour) la linéarité et le degré de récupération, les interférences possibles (y compris les cellules rouges) et les valeurs de référence normales sont presentées. Ses valeurs sont comparées aux résultats obtenus par technique spectrophotométrique. Les problèmes majeurs rencontrés comprenaient des variations unprédictibles de jour en jour pour les echantillons de sérum, la perte de $CO_2$ par les echantillons traversant les tubes de l'analysateur, et des erreurs inexplicables pour le premier echantillon d'une serie. Des méthodes sont suggérées pour surmonter chacune de ces difficultés.

**Beurteilung des Orion SS-20 Ionized Calcium Analyser im klinischen Laboratorium**

Es wird eine Beurteilung des Orion SS-20 Ionized Calcium Analyser präsentiert. Daten werden angeführt bezüglich der Reproduzierbarkeit (von Tag zu Tag und innerhalb einer Messung), der Linearität und der Rekuperation, der möglichen Interferenzen (inklusive roter Zellen), und der normalen Referenzwerte. Die Resultate werden direkt verglichen mit denjenigen, die mit einer spektrophotometrischen Technik erhalten werden können. Aufgetretene Probleme umfassten tägliche Schwankungen des Sensor-Signals auf Serumproben, Verlust von $CO_2$ während des Durchflusses der Proben durch die Geräteschläuche, und unerklärte Fehler bei der ersten Probe einer Serie. Es werden Vorschläge gemacht wie jede dieser Schwierigkeiten bewältigt werden kann.

